# Glatiramer Acetate Treatment in Multiple Sclerosis-Associated Fatigue—Beneficial Effects on Self-Assessment Scales But Not on Molecular Markers

**DOI:** 10.3390/biom11030393

**Published:** 2021-03-07

**Authors:** Oliver Neuhaus, Wolfgang Köhler, Florian Then Bergh, Wolfgang Kristoferitsch, Jürgen Faiss, Thorsten Rosenkranz, Dirk Reske, Robert Patejdl, Hans-Peter Hartung, Uwe K. Zettl

**Affiliations:** 1 Department of Neurology, Klinikum der Heinrich Heine Universität, 40225 Düsseldorf, Germany; hans-peter.hartung@uni-duesseldorf.de; 2 Department of Neurology, SRH Kliniken Landkreis Sigmaringen GmbH, 72488 Sigmaringen, Germany; 3 Department of Neurology, Fachkrankenhaus Hubertusburg, 04779 Wermsdorf, Germany; wolfgang.koehler@medizin.uni-leipzig.de; 4 Department of Neurology, Universität Leipzig, 04103 Leipzig, Germany; florian.thenbergh@medizin.uni-leipzig.de; 5 Department of Neurology, SMZ-Ost-Donauspital, 1220 Vienna, Austria; wolfgang.kristoferitsch@meduniwien.ac.at; 6 Karl Landsteiner Institute for Neuroimmunological and Neurodegenerative Disorders, 1220 Vienna, Austria; 7 Department of Neurology, Asklepios Fachklinikum Teupitz, 15755 Teupitz, Germany; faiss@dsg-berlin.org; 8 German Stroke Society, 10117 Berlin, Germany; 9 Department of Neurology, Asklepios Klinik St. Georg, 20099 Hamburg, Germany; t.rosenkranz@asklepios.com; 10 Department of Neurology, Klinikum der Universität zu Köln, 50937 Cologne, Germany; dirk.reske@lvr.de; 11 Department of Psychiatry, LVR-Klinik Köln, 51109 Cologne, Germany; 12 Department of Physiology, Universitätsmedizin Rostock, 18057 Rostock, Germany; robert.patejdl@uni-rostock.de; 13 Department of Neurology, Universitätsmedizin Rostock, 18057 Rostock, Germany; uwe.zettl@med.uni-rostock.de

**Keywords:** multiple sclerosis, fatigue, glatiramer acetate

## Abstract

Although fatigue is a common symptom in multiple sclerosis (MS), its pathomechanisms are incompletely understood. Glatiramer acetate (GA), an immunomodulatory agent approved for treatment of relapsing-remitting MS (RRMS), possesses unique mechanisms of action and has been shown to exhibit beneficial effects on MS fatigue. The objective of this study was to correlate clinical, neuropsychological, and immunological parameters in RRMS patients with fatigue before and during treatment with GA. In a prospective, open-label, multicenter trial, 30 patients with RRMS and fatigue were treated with GA for 12 months. Inclusion criterion was the presence of fatigue as one of the most frequent and disabling symptoms. Before and during treatment, fatigue was assessed using the Fatigue Severity Scale (FSS), the MS-FSS, and the Modified Fatigue Impact Scale (MFIS). In addition, fatigue and quality of life were assessed using the Visual Analog Scales (VAS). Laboratory assessments included screening of 188 parameters using real-time PCR microarrays followed by further analysis of several cytokines, chemokines, and neurotrophic factors. Fatigue self-assessments were completed in 25 patients. After 12 months of treatment with GA, 13 of these patients improved in all three scales (with the most prominent effects on the MFIS), whereas 5 patients had deteriorated. The remaining 7 patients exhibited inconsistent effects within the three scales. Fatigue and overall quality of life had improved, as assessed via VAS. Laboratory assessments revealed heterogeneous mRNA levels of cytokines, chemokines, and neurotrophic factors. In conclusion, we were not able to correlate clinical and molecular effects of GA in patients with RRMS and fatigue.

## 1. Introduction

Fatigue is a feeling of permanent tiredness at daytime and is a common symptom in all stages of multiple sclerosis (MS) [1,2]. Similar to depression, fatigue is one of the invisible and barely measurable, and thus underrated symptoms, where patients describe enormous impairment of daily activities and quality of life [3]. However, fatigue contributes only indirectly to the Expanded Disability Status Scale (EDSS)—a standard assessment scale of disability in MS [4]—and thus played a minor or no role in previous clinical trials of disease modifying treatments in MS.

The pathomechanisms of fatigue in MS are incompletely understood [1,5]. Suggestions are (i) immunological mechanisms such as overexpression of pro-inflammatory cytokines, (ii) metabolic dysregulation, (iii) diffuse axonal damage, (iv) fatigability due to reduced plasticity of the MS brain, or (v) neuropsychological influences [6,7,8]. Functional MRI studies revealed different areas of cortical activation in fatigued MS patients as compared to non-fatigued patients, indicating different functional patterns in both groups [9,10]. The question if biomolecules—and if yes, which ones—play crucial roles in the pathogenesis of MS fatigue is still unanswered [11]. Various hormones involved in the (chronic or episodic) hypercortisolemia that characterizes MS may contribute to fatigability [12,13]. Furthermore, transmitters involved in attention and arousal, as well as the alteration of their synaptic activity, are discussed [14].

Quantification of MS fatigue remains partially subjective and relies on self-assessment scales. An easy way to “measure” MS fatigue is to ask: “On a scale from 0 (no fatigue) to 10 (maximum imaginable fatigue), where are/were you now/today/the last seven days/the last four weeks?” The answer can be drawn by the patient on a Visual Analog Scale (VAS) [15].

Krupp developed and validated two more distinctive fatigue self-assessment scales addressing the severity of fatigue, the Fatigue Severity Scale (FSS) [16], and the MS-Fatigue Severity Scale (MS-FSS) [17]. Furthermore, the Modified Fatigue Impact Scale (MFIS) assesses the impact of fatigue on daily activities [18]. The self-assessment scales are described in the Supplemental Materials.

According to the incomplete knowledge on MS fatigue pathophysiology, treatment is difficult [1,19]. Apart from behavioral education, such as longer nighttime sleep and regular daytime naps [20], several anti-fatigue agents have been assessed, although they have lacked evidence. Modafinil, approved for treatment of narcolepsy [15], or amantadine, approved for treatment of Parkinson’s disease but with anti-fatigue potencies [17], are frequently used off-label. Based on theories of immune mechanisms related to MS fatigue, the anti-fatigue potential of immunomodulatory drugs such as natalizumab, interferon (IFN) beta, or dimethyl fumarate are under investigation for their anti-fatigue potential [21,22,23,24].

Glatiramer acetate (GA) is an immunomodulatory agent approved to treat relapsing-remitting MS (RRMS) [25,26]. Its development—based on an enormous portion of chance—is a fascinating example of an experimental agent finally approved for treatment in human disease [27]. In the 1970s, researchers at the Weizmann Institute of Science at Rehovot, Israel tried to create an artificial antigen to cause experimental autoimmune encephalitis (EAE) in animal models of MS. To this end, they imitated myelin-basic protein by an artificial protein oligomer mixture of four amino acids, glutamic acid, leucine, alanine, and tyrosine at a defined molar residue ratio. By chance, this protein mixture did not worsen EAE but rather improved disease symptoms [27]. This milestone observation led to human trials in MS where this beneficial effect was confirmed [25]. Finally, GA has been approved for treatment in RRMS.

The mechanisms of action of GA have been investigated intensively and comprise an immune shift from a more pro-inflammatory environment towards anti-inflammatory conditions [28]. Furthermore, neuroprotective properties are discussed [29]. Metz and colleagues observed that GA exhibited beneficial effects on MS fatigue as assessed in patients after six months of treatment with GA [30]; as an outcome measure, the Fatigue Impact Scale (FIS) was used [18].

The aim of this study was to correlate clinical, immunological, and neuropsychological parameters in RRMS patients with fatigue before and during treatment with GA. We intended to define GA-fatigue associated biomolecules.

## 2. Materials and Methods

### 2.1. Patients

In a prospective, open-label, multicenter trial conducted between 2004 and 2007, 30 patients with RRMS and fatigue were treated with GA 20 mg subcutaneously per day for 12 months based on clinical indication at that time. The main inclusion criterion (and the main difference to the patient cohort of Metz’ study) [30] was the presence of fatigue as one of the most frequent and disabling symptoms. To this end, according to Flachenecker and colleagues [31], the following three questions had to be answered with “yes”:

1. Is fatigue one of your three most disabling symptoms?
2. Does fatigue occur daily or on most days?
3. Does fatigue affect your activities at home or at work?

All inclusion and exclusion criteria are given in the Supplementary Materials. Exclusion criteria comprised comorbidities that could make patients susceptible to fatigue, e.g., untreated hypothyroidism.

This study was approved by the Ethics committee of the Heinrich Heine Universität, Düsseldorf, Germany, Study No. 2288, and by all appropriate ethics committees of the participating centers. Written informed consent was obtained from all patients.

### 2.2. Clinical Parameters

EDSS was assessed at baseline and at the last follow-up visit at month 12 [4]. As depression is often closely related to fatigue symptoms [32] and to rule out major depression, the Beck Depression Inventory (BDI) performed at screening had to be 18 or less [33]. The BDI is a self-assessment scale measuring the severity of depression. It ranges between 0 and 63 points. Zero to 10 points, no depression; 11–17 points, mild to moderate depression; and 18–63 points, major depression.

### 2.3. Immunological Parameters

We aimed to correlate MS fatigue with MS immunology at the molecular level. To this end, we screened full-blood mRNA expression of 188 predominantly immunological parameters, as well as neurotrophic factors (Table S1; Supplementary Materials) using real-time PCR microarrays (Applied Biosystems, Foster City, CA, USA) in PAXGene blood samples (Becton Dickinson, Franklin Lakes, NJ, USA), as described previously [34]. Blood sampling was performed at baseline and every three months. We compared baseline to month 9 (higher yield of sampling as compared to month 12) in paired samples obtained from 12 patients.

Detailed qPCR analysis of the following 14 parameters was performed in cDNA samples obtained from 21 patients at baseline and 19 patients at month 9 in order to assess their relationship to MS fatigue:

- Cytokines: interleukin (IL)-4, IL-6, IL-12, IL-17, IFN gamma, tumor necrosis factor (TNF) alpha;
- Chemokines: CCL7, CXCL9, CXCL12;
- Neurotrophic factors: leukemia inhibitory factor (LIF), ciliary neurotrophic factor (CNTF), brain derived neurotrophic factor (BDNF), insulin-like growth factor 1 (IGF1), metallothionin-3 (MT3).

### 2.4. Neuropsychological Parameters

Before and every three months during treatment, fatigue was self-assessed by the patients using several scales:

- Visual Analog Scale (VAS);
- Fatigue Severity Scale (FSS) [16];
- MS-Fatigue Severity Scale (MS-FSS) [17];
- Modified Fatigue Impact Scale (MFIS) [18].

Furthermore, overall quality of life was assessed using an additional VAS.

In our study, we compared baseline to month 12.

### 2.5. Statistical Analysis

Results are given as mean ± standard deviation. Student’s *t*-test was performed for statistical analysis. A *p*-value of <0.05 was accepted to be significant.

## 3. Results

In total, 30 patients were enrolled in our study and 25 patients were available for full analysis. Four patients were excluded because sampling was incomplete and patients were lost to follow-up. One patient was excluded because his BDI at screening exceeded 18, reflecting a major depression.

Baseline and follow-up characteristics are shown in Table 1. Mean BDI sum score was 10.6 ± 4.7 (range 1–16). The study population was heterogeneous, as indicated by the relatively high standard deviations. There were no significant treatment effects on the EDSS or the annualized relapse rate.

### 3.1. GA Did Not Exhibit Significant Effects on the mRNA Level

**Figure 1:**
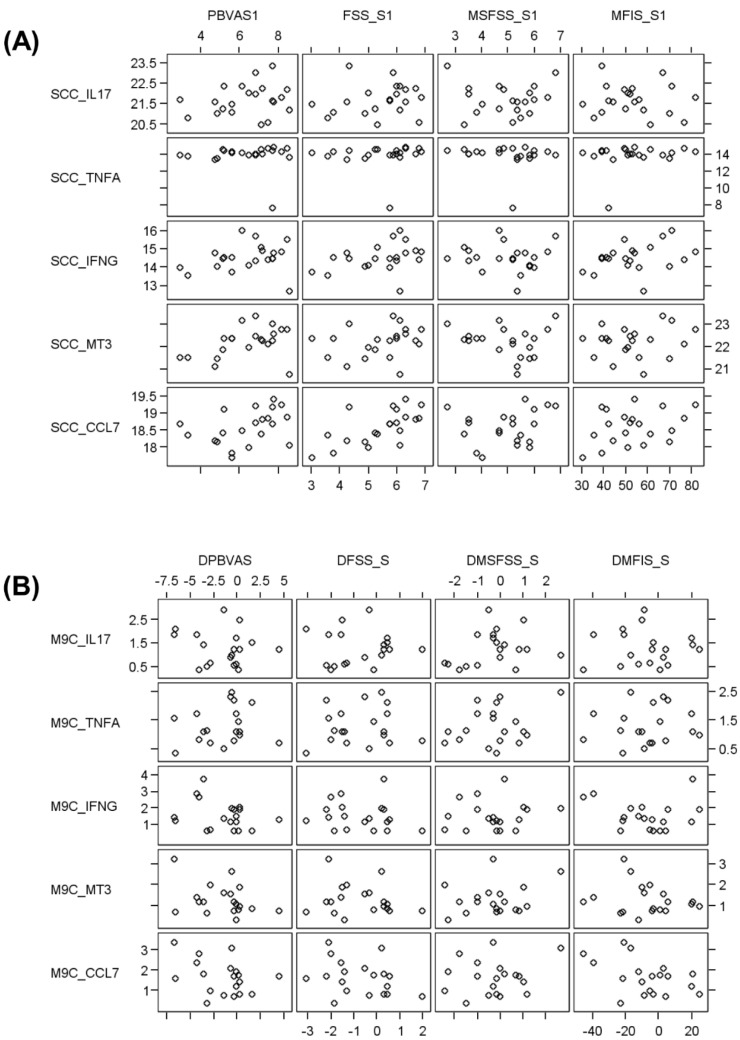
Correlation of Visual Analog Scale (VAS), Fatigue Severity Scale (FSS), MS-Fatigue Severity Scale (MS-FSS), and Modified Fatigue Impact Scale (MFIS) with immunological parameters at (**A**) baseline and (**B**) month 9. Note that the axes in panel (**A**) show the absolute results of the self-assessment scales (*x*-axis) and the amplified cDNA (*y*-axis), whereas the axes in panel (**B**) show the respective individual changes.

Laboratory assessments in samples obtained from 12 patients revealed heterogeneous mRNA levels of cytokines, chemokines, and neurotrophic factors at baseline and month 9, both when assessing all 188 screening parameters in paired samples obtained from 12 patients (Supplemental Materials, Table S1) and when assessing 14 distinct parameters on the cDNA level in samples obtained from 21 patients at baseline, and from 19 patients at month 9. No significant correlations were observed between fatigue scales and immunological parameters. An example of five parameters is illustrated in Figure 1.

### 3.2. GA Partially Improved Fatigue Self-Assessments

Fatigue self-assessments were completed by 25 patients. The mean observation period was 346.4 days. In total, 13 of these 25 patients improved in the three self-assessment scales (FSS, MS-FSS, and MFIS), whereas 5 patients deteriorated. The remaining seven patients exhibited inconsistent effects within the three scales.

**Figure 2:**
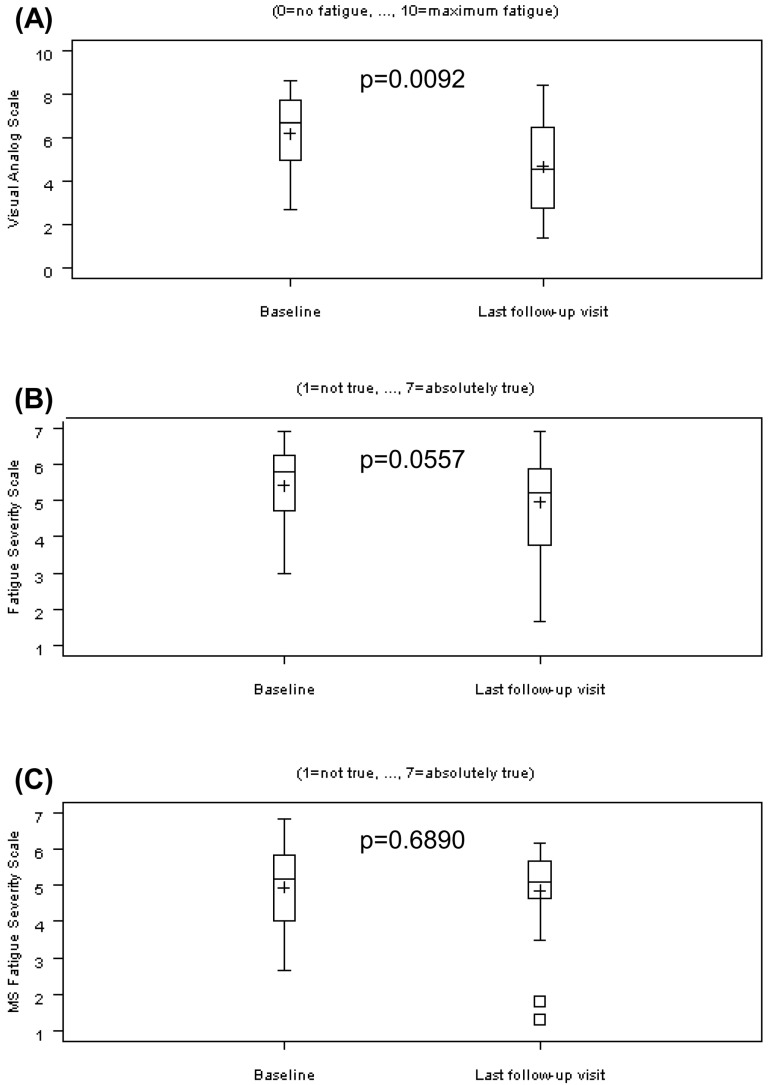
Fatigue self-assessment scales at baseline and last follow-up visit. (**A**) Visual Analog Scale (VAS), range from 0 (no fatigue) to 10 (maximum fatigue); (**B**) Fatigue Severity Scale (FSS), range from one (not true) to seven (absolutely true); (**C**) MS-Fatigue Severity Scale (MS-FSS), range from one (not true) to seven (absolutely true). Results are shown as mean (“+”), median (“―“), and standard errors.

The self-assessment of fatigue on the VAS with a range from 0 (no fatigue) to 10 (maximum fatigue) exhibited a significant decrease from 6.17 ± 1.82 to 4.69 ± 2.12 (*p* = 0.0092; Figure 2A).

The FSS [16] with a range from one (not true) to seven (absolutely true) missed significance and decreased from 5.42 ± 1.08 to 4.96 ± 1.36 (*p* = 0.0557; Figure 2B).

The MS-FSS [17] with a range from 1 (not true) to seven (absolutely true) exhibited no significant effect: minimal decrease from 4.94 ± 1.13 to 4.85 ± 1.21 (*p* = 0.6890; Figure 2C).

**Figure 3:**
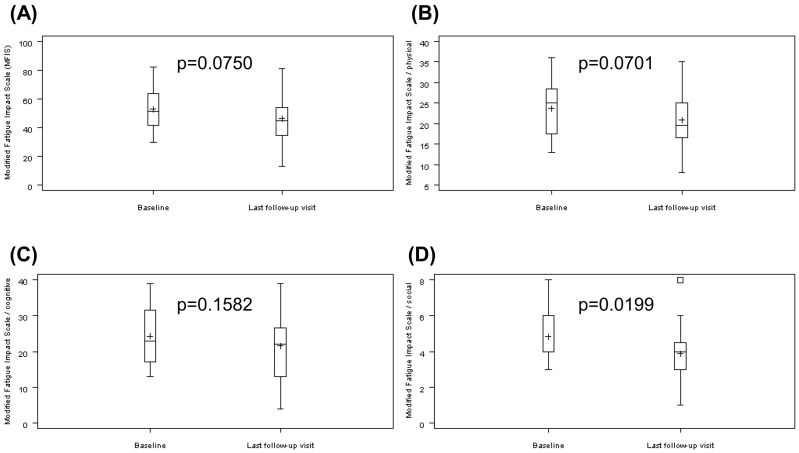
Modified Fatigue Impact Scale (MFIS) at baseline and last follow-up visit. (**A**) MFIS, range from zero (never) to 84 (always); (**B**) MFIS subscore “physical”, range from zero (never) to 36 (always); (**C**) MFIS subscore “cognitive”, range from zero (never) to 40 (always): (**D**) MFIS subscore “social”, range from zero (never) to 8 (always). Results are shown as mean (“+”), median (“―“), and standard errors.

The MFIS [18] with a range from 0 (never) to 84 (always) was not significant assessing the total score: decrease from 52.88 ± 14.22 to 46.29 ± 17.03 (*p* = 0.0750; Figure 3A).

Three subscores of the MFIS were further analyzed:

Subscore “physical” with a range from 0 (never) to 36 (always) missed significance and decreased from 23.75 ± 6.75 to 20.88 ± 6.94 (*p* = 0.0701; Figure 3B).

Subscore “cognitive” with a range from 0 (never) to 40 (always): there were no significant effect: decrease from 24.29 ± 8.02 to 21.54 ± 9.42 (*p* = 0.1582; Figure 3C).

Subscore “social” with a range from 0 (never) to 8 (always): significant improvement was shown: decrease from 4.83 ± 1.49 to 3.88 ± 1.87 (*p* = 0.0199; Figure 3D).

The self-assessment of the overall quality of life on the VAS with a range from 0 (worst imaginable health state) to 100 (best imaginable health state) increased significantly from 57.7 ± 19.80 at baseline to 67.7 ± 16.39 at the last follow-up visit (*p* = 0.0223; data not shown).

## 4. Discussion

This study focused on patients predominantly complaining of fatigue in contrast to motor or sensory MS symptoms. They were predominantly female (80%), relatively old (mean age 41.9 years), and had a low mean EDSS (2.4; Table 1). This observation is not unexpected as patients with higher EDSS probably do not answer question 1 with “yes” (is fatigue one of your three most disabling symptoms?). MS therapists know this type of patients. Even today, with a continuously growing arsenal of approved MS treatments, MS fatigue remains prevalent and its treatment difficult [1,19].

We observed a general yet minor improvement of fatigue levels upon GA treatment in some of the scales used. Significant effects were seen in the VAS and in the subscore “social” of the MFIS. A comparison of the scales is given by Flachenecker et al. [33]. However, we did not observe relevant effects on clinical and immunological parameters. Our goal to correlate biomolecules with effects of GA on fatigue could not be achieved.

The observation of some beneficial effects of GA on fatigue parameters in our special “benign” MS cohort with low EDSS let us speculate that there may be treatment effects of GA beyond immunology; however, we may have observed mere effects of having started treatment at all.

The major limitation of our study is the lack of a control group in an open-label trial. With all caution to interpret self-assessment data, GA was shown to reduce fatigue in several tests. The question if this effect could be confirmed in placebo-controlled trials remains open. To our knowledge, the randomized placebo-controlled approval trials of GA [25]—and other immunomodulatory agents—did not contain fatigue assessments.

Interestingly, and disappointingly, none of the cyto- or chemokine mRNA concentrations showed significant correlation to fatigue scores. While this is in line with previous data [1], we could not confirm a possible role of the proinflammatory cytokine IL-6, discussed by a Dutch group [35]. Our relatively small sample size and the heterogeneity of our patient cohort could explain this observation.

## 5. Conclusions

In summary, this open-label trial indicates that GA may exhibit beneficial effects in some fatigued RRMS patients. The mechanisms of these effects remain elusive; they are not reflected by full blood cytokine mRNA expression. Further controlled trials are highly warranted.

## Figures and Tables

**Table 1:** Baseline and follow-up characteristics (*n* = 25).

Parameter	Baseline Visit	Last Follow-Up Visit
Age (years)	41.9 ± 8.0 (22.5–55.4)	
Gender	f:m = 80:20	
Time since diagnosis (months)	56.8 ± 69.3 (0.4–270.6)	
Number of relapses since diagnosis	4.8 ± 4.3	
Number of relapses in previous 2 years	1.8 ± 1.0	
Number of relapses during therapy		0.7 ± 0.7
Annualized relapse rate	0.9	0.8 (n.s.)
EDSS	2.4 ± 1.1 (1.0–4.5)	2.5 ± 0.9 (1.0–4.0, n.s.)
BDI	10.6 ± 4.7 (1–16)	

Results are given as mean ± standard deviation (range). BDI: Beck Depression Inventory [33]; EDSS: Expanded Disability Status Scale [4]; n.s.: not significant.

## Data Availability

Data supporting reported results can be obtained at the corresponding author.

## Supplementary Materials

The following are available online at https://www.mdpi.com/2218-273X/11/3/393/s1, fatigue self-assessment scales, inclusion and exclusion criteria of our study, Table S1: transcripts analyzed using real-time PCR microarrays.
