# Supplementary material for: Glatiramer Acetate Treatment in Multiple Sclerosis-Associated Fatigue—Beneficial Effects on Self-Assessment Scales But Not on Molecular Markers
Source: Biomolecules. 2021 Mar 7;11(3):393. doi: 10.3390/biom11030393 (PMC8002075; doi:10.3390/biom11030393)
Supplement: Supplementary file 1 [file biomolecules-11-00393-s001.pdf]

Supplementary Material. Fatigue self-assessment scales

Fatigue Severity Scale (FSS) [16]

Nine items. The score ranges from 1 = not true to 7 = absolutely true. The overall score is given as the mean of the nine items (range 1 to 7). The higher the score is, the more severe is the fatigue.

1. My motivation is lower when I am fatigued.
2. Exercise brings on my fatigue.
3. I am easily fatigued.
4. Fatigue interferes with my physical functioning.
5. Fatigue causes frequent problems to me.
6. My fatigue prevents sustained physical functioning.
7. Fatigue interferes with carrying out certain duties and responsibilities.
8. Fatigue is among my three most disabling symptoms.
9. Fatigue interferes with my work, my family, or social life.

MS-Fatigue Severity Scale (MS-FSS) [17]

Six items. The score ranges from 1 = not true to 7 = absolutely true. The overall score is given as the mean of the six items (range 1 to 7). The higher the score is, the more severe is the fatigue.

1. Heat worsens my fatigue.
2. Long periods of inactivity worsen the fatigue.
3. Stress worsens the fatigue.
4. Depressive mood worsens the fatigue.
5. Cool temperatures improve my fatigue.
6. Positive experiences improve my fatigue.

Modified Fatigue Impact Scale (MFIS) [18]

Twenty-one items assessing physical, cognitive, social and total fatigue. The overall score is given as the sum of the responses to 21 single questions (0 = never; 1 = rarely; 2 = sometimes; 3 = frequently; 4 =always; range 0 to 84). The higher the score is, the higher is the impact of fatigue on daily activities.

The overall MFIS score can be divided into the three subscores physical (nine questions), cognitive (ten questions) and social (two questions).

During the last four weeks, because of my fatigue…

1. … I was less alert
2. … I had difficulties pursuing things for longer time
3. … I was not able to think clearly
4. … I was inapt and uncoordinated
5. … I was forgetful
6. … I had to limitate my physical activity
7. … I was barely motivated to do things associated with physical effort
8. … I was barely motivated to take part in social activities
9. … I was limitated to do things outside my home
10. … I had difficulties to maintain efforts over longer time periods
11. … I had difficulties to make decisions
12. … I was barely motivated to do things I had to concentrate on
13. … I felt weak
14. … I did not feel well physically
15. … I had difficulties to finish things I had to concentrate on
16. … I had difficulties to hold my thoughts together at home or at work
17. … I was not able to finish things requiring physical effort
18. … my thinking was slowed
19. … I had difficulties to concentrate
20. … I limited my physical activities
21. … I needed more or longer breaks

Supplementary Material. Inclusion and exclusion criteria of our study

Inclusion criteria

- Patients with relapsing-remitting multiple sclerosis (MS); therapy with glatiramer acetate (GA) is indicated according to treatment guidelines
- Presence of fatigue as one of the most frequent and disabling symptoms. The following three questions had to be answered with “yes” [31]:

1. Is fatigue one of your three most disabling symptoms?
2. Does fatigue occur daily or at most days?
3. Does fatigue affect your activities at home or at work?

- Signed consent form

Exclusion criteria

- Previous treatment with GA
- Treatment with other immunomodulatory agents within two months before baseline
- Treatment with other immunosuppressive agents within six months before baseline
- MS relapse within six weeks before baseline
- Treatment with steroids within four weeks before baseline
- Pregnancy or breast feeding within six months before baseline
- Major depression (Beck Depression Inventory [BDI] > 18) [33]
- Untreated hypothyroidism
- Symptomatic treatment of MS with sedating agents whose dose can increase MS fatigue (such as baclofen or carbamazepine)

Table S1. Transcripts analyzed using real-time PCR microarrays (in alphabetical order)

No	Abbreviation	Transcript
1	ARTN	Artemin (neurotrophic factor)
2	BDNF	Brain derived neurotrophic factor
3	BLR-1	Blue-light regulator 1 (transcription factor)
4	BSG	Basigin = CD147
5	CBLN1	Cerebellin-1
6	CCL1	Chemokine CCL1
7	CCL5	Chemokine CCL5
8	CCL7	Chemokine CCL7
9	CCL11	Chemokine CCL11
10	CCL17	Chemokine CCL17
11	CCL21	Chemokine CCL21
12	CCR1	Chemokine receptor CCR1
13	CCR2	Chemokine receptor CCR2
14	CCR3	Chemokine receptor CCR3
15	CCR4	Chemokine receptor CCR4
16	CCR5	Chemokine receptor CCR5
17	CCR6	Chemokine receptor CCR6
18	CCR7	Chemokine receptor CCR7
19	CCR8	Chemokine receptor CCR8
20	CCR9	Chemokine receptor CCR9
21	CCR10	Chemokine receptor CCR10
22	CD4	CD4
23	CD7	CD7
24	CD8A	CD8A
25	CD14	CD14
26	CD19	CD19
27	CD22	CD22
28	CD28	CD28
29	CD38	CD38
30	CD40	CD40
31	CD44	CD44
32	CD79B	CD79B
33	CD86	CD86
34	CD247	CD247
35	CEBPB	Cebpb (transcription factor)
36	CNTF; ZFP91	Ciliary neurotrophic factor
37	CNTFR1	Ciliary neurtrophic factor receptor 1
38	CRHBP	Corticotropin releasing hormone binding protein
39	CRHR1	Corticotropin releasing hormone receptor 1
40	CRHR2	Corticotropin releasing hormone receptor 2
41	CSF2	Colony stimulating factor 2 (= granulocyte macrophage colony stimulating factor)
42	CTLA4	Cytotoxic T-lymphocyte associated protein 4
43	CXCR3	Chemokine receptor CXCR3
44	CXCR4	Chemokine receptor CXCR4
45	CXCL3	Chemokine CXCL3
46	CXCL5	Chemokine CXCL5
47	CXCL9	Chemokine CXCL9
48	CXCL10	Chemokine CXCL10
49	CXCL11	Chemokine CXCL11
50	CXCL12	Chemokine CXCL12
51	CXCL13	Chemokine CXCL13
52	CX3CR1	Chemokine receptor CX3CR1
53	FAS	Fas = CD95
54	FASLG	Fas ligand
55	FCGR1A	Fc gamma receptor 1A
56	FCGR2A	Fc gamma receptor 2A
57	FCGR3A	Fc gamma receptor 3A
58	FGF2	Fibroblast growth factor 2

59	FGF9	Fibroblast growth factor 9
60	FGFR1	Fibroblast growth factor receptor 1
61	FOXP3	Forkhead box P3
62	FRS2	Fibroblast growth factor receptor substrate 2
63	FRS3	Fibroblast growth factor receptor substrate 3
64	FUS	FUS (Fused in sarcoma)
65	GATA3	GATA binding protein 3
66	GDNF	Glial cell line-derived neurotrophic factor
67	GFRA1	GDNF family receptor alpha 1
68	GRFA2	GDNF family receptor alpha 2
69	GFRA3	GDNF family receptor alpha 3
70	GFI1	GF11 (transcription regulator)
71	GMFB	Glia maturation factor beta
72	GMFG	Glia maturation factor gamma
73	GPR44	G protein-coupled receptor 44
74	GZMA	Granzyme alpha
75	GZMB	Granzyme beta
76	HAVCR2	Hepatitis A virus cellular receptor 2
77	HLA-A	Human leukocyte antigen A
78	ICAM1	Intercellular adhesion molecule 1
79	ICOS	Inducible T-cell costimulator
80	IFNG	Interferon gamma
81	IFNGR1	Interferon gamma receptor 1
82	IGF1	Insulin-like growth factor 1
83	IGSF6	Ig superfamily 6
84	IGHA1	Immunoglobulin heavy constant alpha 1
85	IGHD	Immunoglobulin heavy constant delta
86	IGHG1	Immunoglobulin heavy constant gamma 1
87	IGHM	Immunoglobulin heavy constant mu
88	IGKC; IGKV1-5	Immunoglobulin kappa constant chain; immunoglobulin kappa variant chains 1-5
89	IGL@; CKAP2	Immunoglobulin light chain
90	IL1B	Interleukin 1B
91	IL1R1	Interleukin 1 receptor 1
92	IL1R2	Interleukin 1 receptor 2
93	IL2	Interleukin 2
94	IL2RA	Interleukin 2 receptor A
95	IL2RB	Interleukin 2 receptor B
96	IL4	Interleukin 4
97	IL4R	Interleukin 4 receptor
98	IL5	Interleukin 5
99	IL6	Interleukin 6
100	IL7	Interleukin 7
101	IL7R	Interleukin 7 receptor
102	IL8RA	Interleukin 8 receptor A
103	IL8RB	Interleukin 8 receptor B
104	IL9	Interleukin 9
105	IL10	Interleukin 10
106	IL12A	Interleukin 12A
107	IL12B	Interleukin 12B
108	IL12RB2	Interleukin 12 receptor B2
109	IL13	Interleukin 13
110	IL13RA1	Interleukin 13 receptor A1
111	IL15	Interleukin 15
112	IL17A	Interleukin 17A
113	IL18	Interleukin 18
114	IL18R1	Interleukin 18 receptor 1
115	IL23A	Interleukin 23A
116	IRF1	Interferon regulatory factor 1
117	IRF4	Interferon regulatory factor 4
118	ITGB2	Integrin beta 2
119	JAK1	Janus kinase 1
120	KCNA3	Voltage-gated potassium channel Kv1.3

121	KCNN4	Voltage-gated potassium channel Kca3.1
122	LAG3	Lymphocyte activation gene 3
123	LIF	Leukemia inhibitory factor
124	LTA	Leukotriene alpha
125	MAF	Musculoaponeurotic fibrosarcoma (transcription factor)
126	MAGED1	Melanoma associated antigen D1
127	MCTS1	Monocyaboxylate transporters 1
128	MEF2C	Myocyte enhancer factor 2C (transcription factor)
129	MKI67	Marker of proliferation Ki-67
130	MS4A1	Membrane spanning 4-domains A1 (encodes CD20)
131	MT3	Metallothionin-3
132	MYD88	Myeloid differentiation primary response gene 88
133	NCAM1	Neural cell adhesion molecule 1
134	NELL1	Neural epidermal growth factor-like 1 protein
135	NF1	Neurotrophic factor 1
136	NFATC1	Nuclear factor of activated T cells 1
137	NFATC2	Nuclear factor of activated T cells 2
138	NFATC2IP	Nuclear factor of activated T cells 2ip
139	NGFB	Nerve growth factor beta
140	NGFR	Nerve growth factor receptor
141	NGFRAP1	Nerve growth factor receptor associated protein 1
142	NR1/2	NMDA receptor 1/2
143	NRG1	Neuregulin 1
144	NRG2	Neuregulin 2
145	NTF3	Neurotrophic factor 3
146	NTF5	Neurotrophic factor 5
147	NTRK1	NTRK1 = tyrosine kinase receptor A
148	NTRK2	NTRK2 = tyrosine kinase receptor 2
149	PAX5	PAX5 (transcription factor)
150	PCGF2	Polycomb group factor 2
151	PRF1	Perforin 1
152	PSPN	Persephin
153	PTGER2	Prostaglandin E receptor 2
154	PTPRC	Protein tyrosine phosphatase receptor type C
155	SDC1	Syndecan 1
156	SLC3A2	SLC3A2 = heavy-chain subunit CD98 of the cysteine/glutamate transporter system
157	SMAD7	Smad 7
158	SOCS1	Suppressor of cytokine signaling 1
159	SOCS5	Suppressor of cytokine signaling 5
160	STAT1	Signal transducer and activator of transcription 1
161	STAT3	Signal transducer and activator of transcription 3
162	STAT4	Signal transducer and activator of transcription 4
163	STAT6	Signal transducer and activator of transcription 6
164	TBX21	T-box transcription factor 21
165	TFG	TRK-fused gene
166	TGFA	Transforming growth factor alpha
167	TGFB1	Transforming growth factor beta 1
168	TGFB2	Transforming growth factor beta 2
169	TGFBR1	Transforming growth factor beta receptor 1
170	TGFBR2	Transforming growth factor beta receptor 2
171	TLR1	Toll-like receptor 1
172	TLR2	Toll-like receptor 2
173	TLR3	Toll-like receptor 3
174	TLR4	Toll-like receptor 4
175	TLR5	Toll-like receptor 5
176	TLR6	Toll-like receptor 6
177	TLR7	Toll-like receptor 7
178	TLR8	Toll-like receptor 8
179	TLR9	Toll-like receptor 9
180	TLR10	Toll-like receptor 10
181	TMED1	Transmembrane emb24 domain 1
182	TNF	Tumor necrosis factor

183	TNFRSF7	Tumor necrosis factor receptor SF7
184	TNFRSF8	Tumor necrosis factor receptor SF8
185	TNFRSF13C	Tumor necrosis factor receptor SF13C
186	TRO	Trophinin
187	VGF	Vascular growth factor
188	XCR1	Chemokine receptor XCR1
